# Shape Adjustment and Experimental Study of a Shape Memory Cable (SMC) Structure

**DOI:** 10.3390/ma16093476

**Published:** 2023-04-29

**Authors:** Fengqun Pan, Xiangjun Jiang, Jingli Du, Jia Liu, Yesen Fan, Wusong Zou

**Affiliations:** 1 Key Laboratory of Electronic Equipment Structure Design, Xidian University, Xi’an 710071, China; fqpan@stu.xidian.edu.cn (F.P.); jldu@mail.xidian.edu.cn (J.D.); liuzjia@stu.xidian.edu.cn (J.L.); 2 China Academy of Space Technology (Xi’an), Xi’an 710100, China; fanyesen@126.com; 3 AVIC Leihua Electronic Technology Research Institute, Wuxi 214063, China; zouws@avic.com

**Keywords:** shape memory cable net structure, shape adjustment, thermal deformation, experimental study, surface accuracy

## Abstract

Large deployable cable net antennas have attracted extensive attention worldwide because of their simple structure and high storage ratio. The cable net structure is affected by long exposure in a harsh space environment during satellite operation, resulting in large thermal deformation and stress relaxation, which leads to a degradation of antenna performance. To address the thermal deformation of the cable net structure, a shape memory cable (SMC) net structure model was proposed with surface accuracy as the research objective. Specifically, we aimed to utilize its phase transition characteristics to adjust the thermal deformation of cable net structure and improve its surface accuracy. A shape memory cable net structure model with a diameter of 2.2 m was built, and a normal temperature experiment and high- and low-temperature experiments were carried out. High- and low-temperature test refers to environmental simulation testing of shape memory cable net structures under high- and low-temperature conditions. This was done to determine whether the adjustment method for surface accuracy meets the requirements. The results showed that the shape memory alloy wire has a relatively stable ability to adjust the surface accuracy of the cable net structure at room temperature. During temperature cycling, the thermal deformation of the shape memory cable net structure is slight, and the surface accuracy is good. Compared with ordinary cable net structures, the shape memory cable net structure has improved surface accuracy by 44.4% and 45.2% at high and low temperatures, respectively. This proved the effectiveness of the method for adjusting surface accuracy. These experimental results offer guiding significance for engineering applications.

## 1. Introduction

Spaceborne deployable antennas are widely used in science, technology, military and other fields such as navigation, mobile communication, electronic reconnaissance and remote sensing. They are important components in a satellite system. A deployable cable net antenna with a large peripheral truss has a simple structure and large compression ratio, and its antenna diameter can reach 150 m. It is one of the most typical structural forms of spaceborne antennas. The peripheral truss deployable antenna has high thermal sensitivity. The accuracy of the antenna profile has a great impact on its frequency and pointing accuracy, and any small deformations can significantly reduce the electrical performance of the antenna [1]. Other studies have shown that the atomic oxygen environment and ultraviolet radiation have important effects on the mechanical properties of polyimide and other polymers [2]. During the operation of spaceborne antennas in orbit, due to the drastic change in the short-term temperature field and the influence of long-term. relaxation, the cable net structure can suffer large deformations, which seriously affect the surface accuracy of the reflector.

Many scholars have carried out research on solar radiation and temperature fields in orbit. Li et al. calculated the transient temperature field of a solar cell array in a low Earth orbit, revealing the cause of thermally induced vibration [3]. Shen et al. analysed the dynamic behaviour of an AstroMesh antenna under the impact of solar flux. Their research results showed that the thermal deformation of the AstroMesh antenna was mainly caused by the temperature change along the axis of the rod, while the temperature gradient on the cross-section of the rod was not the main reason [4]. Zhang et al. predicted the in-orbit thermal deformation of a high-resolution satellite camera [5]. Du et al. discussed the shape adjustment of cable net antennas and proposed a method for shape-adjusting the cable net structures based on optimization, which effectively reduced the profile error and was easy to solve [6]. Guo et al. proposed a thermal compensation and precompensation design for an antenna and carried out a prestressed design according to the temperature load [7]. Yang et al. designed a typical AstroMesh reflector structure to stabilize the surface shape and proposed a method to precontrol the in-orbit thermal deformation of the grid reflector to reduce the thermal deformation of the reflector in the entire temperature range [8]. Nie et al. analysed the coupling effect of a truss and cable net in the thermal deformation process, reduced the thermal deformation error by improving the optimization model, and reduced the in-orbit profile error of the reflector by optimizing the thermal structure model [9,10].

Various studies have been carried out on shape memory alloys as control and drive actuators in aerospace applications [11,12,13,14]. Scientists at the Ohio State University (OSU) in the United States made smart materials into piezoelectric actuators to provide different antenna radiation patterns by controlling the deformation of the reflector [15]. Saravanan et al. proposed an intelligent truss structure (VGT), used a genetic algorithm to search for the optimal solution, and obtained the optimal configuration scheme for the number and position of actuators, thus optimizing the reflector error of a Doppler antenna [16]. Peng made a shape memory alloy wire into an actuator and produced the expected tension according to the design, which was used to control the shape adjustment process of inflatable synthetic aperture radar (SAR) antenna and achieve high control accuracy [17]. Due to the special phase transformation behaviour of smart materials, SMAs have also been applied in the aerospace field, such as deformed wings and self-expanding solar sails. [18]. Intelligent materials have also been applied in optics and acoustics. Wang et al. have manufactured membrane constrained acoustic metamaterial. By adjusting the position of the rod, the central position of the membrane can be constrained to achieve different vibration modes [19]. Its principle is similar to the shape memory effect of SMA. Valagianopoulos et al. proposed the bistable phenomenon of optically driven memory elements relative to the incident angle and demonstrated their practicality [20]. The shape memory effect of SMA driven by temperature can also be used for switches or memories in electromagnetic systems. Research by Marinopoulou and Katakalos has shown that iron-based SMAs have good recovery stress during cyclic testing [21]. Wang et al. studied the adjustment method of smart materials to the precision of the cable mesh structure profile, made piezoelectric ceramics into actuators, and controlled the driving force of the actuators through the adjustment of voltage to adjust the shape of the cable mesh structure reflector [22]. Song et al. used piezoelectric materials as actuators to improve the surface accuracy of antenna reflectors [23]. Jiang et al. proposed a shape memory cable net structure, studied the optimization process of surface accuracy, and verified its effectiveness through simulations [24,25]. Furuya et al. studied the performance of actuators on shape control, analysed the data statistics method of space truss deformation, used active elements to estimate the performance of the static shape control of antenna trusses, and used genetic algorithms to obtain the optimal placement of actuators [26].

The above research mainly optimizes the cable net structure in two ways. One is to improve the calculation method to improve the surface accuracy through compensation. Due to the complexity of the space environment, this method cannot completely eliminate the influence of thermal deformation on the surface accuracy. The second is to make intelligent materials into drivers to adjust the profile accuracy. This method promotes the development of intelligent space truss structures. However, the process of using smart materials as drivers requires an external high-voltage power supply, which increases the weight of the whole structure. At the same time, it is difficult to achieve a long-term high power supply during the operation of the antenna in orbit. To solve the above problems, shape memory alloy wire was embedded into a cable net structure to form a shape memory cable (SMC) net structure. The shape memory alloy phase transformation was used to adjust the profile accuracy. The cable net structure undergoes significant deformation with temperature changes, resulting in a decrease in surface accuracy. The pre-compensation optimization method proposed by scholars cannot fundamentally solve the thermal deformation problem of cable net structures. Shape memory alloy wires generate significant strain and stress during the phase transformation process. It is thus necessary to compensate for the thermal deformation caused by temperature changes in the cable net structure.

The calculations for addressing these challenges begin with selecting a communication antenna with a frequency of 300 MHz as the research object. The required antenna diameter can be calculated from the wavelength and gain.

The wavelength can be calculated by the following equation:

(1) $$\lambda =\frac{c}{f},$$

where λ is the wavelength, c is the wave speed, and f is the frequency.

The relationship between gain and wavelength can be expressed as:

(2) $$G=\frac{4\pi A_{e}}{\lambda^{2}},$$

where G is the gain and $A_{e}$ is the effective area; thus, the relationship between the effective area of the reflector antenna and the diameter can be shown in Equation (3):

(3) $$A_{e}=\frac{\pi D^{2}\eta }{4},$$

In the formula, D is the diameter of the antenna and η is the efficiency of the antenna. The diameter of the antenna can be calculated from Equations (2) and (3), and is shown in Equation (4):

(4) $$D=\frac{\lambda }{\pi }\sqrt{\frac{G}{\eta }}.$$

A shape memory cable net structure with a diameter of 2.2 m was built, and experimental research was carried out. First, the external power supply was used to actively adjust the reflective surface of the shape memory cable net structure to verify the adjustment effect of the shape memory alloy on the reflective surface. Then, the power supply was removed and the structure passively adjusted with the change in space ambient temperature to reduce the overall weight. The experimental results showed that this method can effectively improve the surface accuracy of cable net structures.

## 2. Materials and Methods

### 2.1. Model of the SMC Structure

#### 2.1.1. Constitutive Model

**Figure 1:**
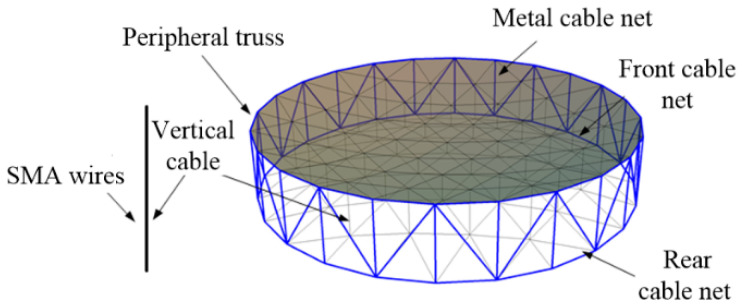
Shape memory cable net structure.

Shape memory alloy wires are embedded into the vertical cable of the cable net structure, and an active component is integrated to form the shape memory cable (SMC) net structure [24], as shown in Figure 1. Space thermal radiation is used to make shape memory alloys produce phase transformation and inverse phase transformation, adaptively change the structural characteristics, realize the adjustment of the reflective surface of the cable mesh structure, and improve the on-orbit retention of the surface accuracy. The shape memory cable net structure is applicable to a wide range of temperatures, and the temperature fields of spaceborne antennas in different orbits are quite different. To make the phase transition temperature meet the design requirements, it is necessary to select an appropriate shape memory alloy according to the actual working conditions.

The truss deformation is not considered in the calculations, and the constitutive model of the shape memory cable is established first. Based on the Brinson model [27], a one-dimensional constitutive model of the shape memory cable can be expressed as

(5) $$\sigma^{s}-\sigma_{0}^{s}=E^{s}\left(\xi \right)\varepsilon^{s}-E^{s}\left(\xi_{0}\right)\varepsilon_{0}^{s}+ϒ\left(\xi \right)\xi -ϒ\left(\xi_{0}\right)\xi_{0}+H\left(T^{s}-T_{0}^{s}\right),$$

where $\sigma^{s}$ and ξ are the stress and martensite volume fraction of the shape memory alloy, respectively. $E^{s}\left(\xi \right)$ is the elastic modulus, $ϒ\left(\xi \right)$ is the phase transformation coefficient, and H is the thermoelastic coefficient. $\sigma_{0}^{s}$, $\varepsilon_{0}^{s}$, $T_{0}^{s}$ and $\xi_{0}$ are the initial states of the memory alloy.

The constitutive equation is expressed in incremental form between the force, temperature and deformation of the shape memory alloy wire to solve the stiffness equation of the cable element.

(6) $$F^{s}-F_{0}^{s}=E^{s}(\xi )A^{s}\left(\frac{x}{L^{s}}-\frac{x}{L_{0}^{s}}\right)+\left[ϒ(\xi )\xi -ϒ(\xi_{0})\xi_{0}+H(T^{s}-T_{0}{}^{s})-E^{s}(\xi_{0})\varepsilon_{0}^{s}\right]A^{s}\frac{x}{L^{s}},$$

where $F^{s}=A^{s}\sigma^{s}\frac{x}{L^{s}}$ is the force vector of the SMA cable element, $F_{0}^{s}$ is the initial force vector of the SMA cable element, $A^{s}$ is the cross-sectional area of the SMA cable element, $L_{0}^{s}$ is the unstressed initial length of the SMA cable element, $L^{s}$ is the real-time length of the SMA cable element after deformation, and $x/L^{s}$ is the nodal position unit vector of the SMA cable element.

By setting

(7) $$\Phi =ϒ(\xi )\xi -ϒ(\xi_{0})\xi_{0}+H(T^{s}-T_{0}{}^{s})-E^{s}(\xi_{0})\varepsilon_{0},$$

further solutions can be obtained as follows:

(8) $$\Delta F^{s}=\frac{k_{1}}{k_{3}}\Delta x+\frac{k_{2}}{k_{3}}\Delta T=K_{D}^{s}\Delta x+K_{T}^{s}\Delta T,$$

Parameters $k_{1}$, $k_{2}$ and $k_{3}$ are expressed as:

(9) $$\left\{\begin{array}{l} k_{1}=\frac{E^{s}A^{s}}{L^{s}}\left(\frac{xx^{T}}{L^{s}{}^{2}}+\frac{L^{s}-L_{0}^{s}}{L_{0}^{s}}I_{3\times 3}\right)+\frac{A^{s}\Phi }{L^{s}}\left(I_{3\times 3}-\frac{xx^{T}}{L^{s}{}^{2}}\right)\\ k_{2}=A^{s}\left(\frac{x}{L_{0}^{s}}-\frac{x}{L^{s}}\right)\frac{dE^{s}}{d\xi }\frac{d\xi }{dT^{s}}+\frac{A^{s}x}{L^{s}}\left(\xi \frac{dϒ}{dE^{s}}\frac{dE^{s}}{d\xi }\frac{d\xi }{dT^{s}}+ϒ\frac{d\xi }{dT^{s}}+H\right)\\ k_{3}=I_{3\times 3}-\frac{xx^{T}}{L^{s}{}^{2}}\left(\frac{L^{s}-L_{0}^{s}}{L_{0}^{s}}\frac{dE^{s}}{d\xi }\frac{d\xi }{d\sigma^{s}}+\xi \frac{dϒ}{dE^{s}}\frac{dE^{s}}{d\xi }\frac{d\xi }{d\sigma^{s}\;}+ϒ\frac{d\xi }{d\sigma^{s}}\right) \end{array}\right.,$$

where $K_{D}^{s}$ and $K_{T}^{s}$ are the stiffness matrices that reflect the relationship between the force and the deformation and between the force and the temperature of the SMA cable element, respectively.

Since the polymer cable segment must always be in the elastic stage, its one-dimensional constitutive equation is

(10) $$\sigma^{c}=E^{c}\left(\varepsilon^{c}-\alpha^{c}T^{c}\right),$$

where $E^{c}$ and $\alpha^{c}$ are the elastic modulus and thermal expansion coefficient of the polymer and $\varepsilon^{c}$ and $T^{c}$ are the strain and temperature of the polymer, respectively.

The constitutive equation is expressed as the incremental relationship between force, temperature and deformation to establish the finite element equation:

(11) $$F^{c}{=A}^{c}\sigma \frac{x}{L^{c}}=\frac{E^{c}A^{c}}{L_{0}^{c}}\left(L^{c}-L_{0}^{c}\right)\frac{x}{L^{c}}-E^{c}A^{c}\alpha \left(T-T_{0}\right)\frac{x}{L^{c}},$$

where $A^{c}$ is the cross-sectional area of the polymer cable element, which is further arranged as follows:

(12) $$\Delta F^{c}=K_{D}^{c}\Delta x+K_{T}^{c}\Delta T,$$

Among them,

(13) $$\left\{\begin{array}{l} K_{D}^{c}=\frac{E^{c}A^{c}}{L^{c}}\left[1+\alpha \left(T-T_{0}\right)\right]\left(\frac{2xx^{T}}{L^{c2}}-\frac{1}{L^{c}}\right)+\frac{E^{c}A^{c}}{L^{c}}\frac{L^{c}}{L_{0}^{c}}\left(\frac{1}{L^{c}}-\frac{xx^{T}}{L^{c2}}\right)\\ K_{T}^{c}{=-E}^{c}A^{c}\alpha \frac{x}{L^{c}} \end{array}\right.,$$

where $K_{D}^{c}$ and $K_{T}^{c}$ are the stiffness matrices that reflect the relationship between the force and the deformation and between the force and the temperature of the polymer cable element, respectively.

The stiffness equations of the shape memory cable and polymer cable segment are set by the finite element method, and then the finite element model of the shape memory cable net structure can be expressed as [24]:

(14) $$\left\{\begin{array}{l} \Delta F^{s}=K_{D}^{s}\Delta X+K_{T}^{s}\Delta T^{s}\\ \Delta F^{c}=K_{D}^{c}\Delta X+K_{T}^{c}\Delta T^{c} \end{array}\right.,$$

(15) $$\Delta F=K_{D}\Delta d+K_{T}\Delta T$$

where $K_{D}^{s}$ and $K_{T}^{s}$ are the stiffness matrices of the relationship between the force and deformation and the relationship between the force and temperature of the shape memory alloy wires, respectively. $K_{D}^{c}$ and $K_{T}^{c}$ are stiffness matrices of the relationship between polymer force and deformation and the relationship between polymer force and temperature, respectively. ΔF is the increment of the external load vector of the cable elements, and Δd and ΔT are the change vectors of the displacement and temperature of each node after the assembly and satisfy the formula Δd=ΔX. $K_{D}$ and $K_{T}$ are the stiffness matrices after assembly, and the expressions are

(16) $$\left\{\begin{array}{l} K_{D}=K_{D}^{s}+K_{D}^{c}\\ K_{T}=K_{T}^{s}+K_{T}^{c} \end{array}\right.,$$

#### 2.1.2. Reflector Adjustment Method

**Figure 2:**
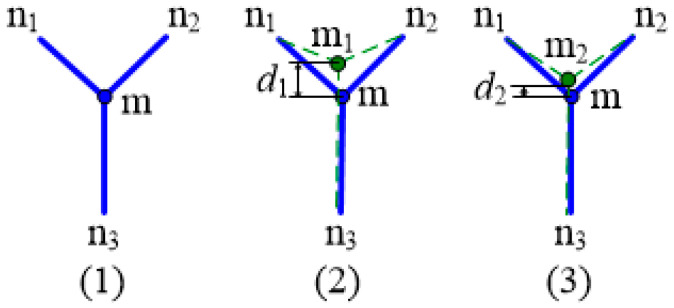
Node position is offset downwards.

**Figure 3:**
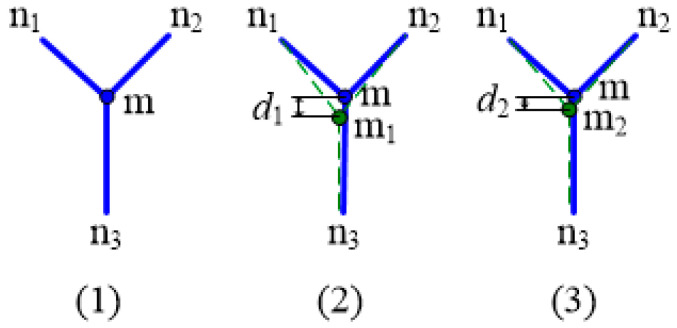
Node position is offset upwards.

The node position of the reflecting surface can be adjusted by using the phase transformation characteristics of the shape memory alloy wire. The adjustment process is shown in Figure 2. Figure 2(1), Figure 2(2) and Figure 2(3) respectively represent the initial state, the state after thermal deformation, and the optimized state. Node M is a node on the reflecting surface, connected to the SMA vertical cable. Taking node m as an example, the reflecting surface of the cable net structure is an ideal paraboloid, and the initial position of the node is shown in Figure 2(1) and Figure 3(1). Under the influence of temperature and other factors, node m deviates from the initial position and moves upwards to m_1_, with an offset distance of *d*_1_, as shown in Figure 2(2). The shape memory alloy wire is heated. When the temperature reaches the temperature at which the austenite phase transformation begins, the shape memory alloy undergoes reversed-phase transformation, the strain decreases, and the reflecting surface node connected with it moves downwards. Node m moves to m_2_, as shown in Figure 2(3), and the distance from the initial position is *d*_2_. *d*_2_ is less than *d*_1_. After adjustment, the node m2 is closer to the initial position m. All nodes of the reflecting surface may be simultaneously adjusted to make the adjusted reflecting surface closer to the initial ideal parabolic surface, and improve the accuracy of the surface.

Figure 3(1), Figure 3(2) and Figure 3(3) respectively represent the initial state, the state after thermal deformation, and the optimized state. Similarly, when node m moves downwards from the initial position, it is shown in Figure 3(2). At this time, the shape memory alloy wire is cooled. When the temperature reaches the temperature at which the martensitic transformation begins, the shape memory alloy undergoes transformation, the strain increases, and the reflecting surface node connected with it moves up. Node m moves to m_2_, as shown in Figure 3(3), and the distance from the initial position is *d*_2_. By simultaneously adjusting all nodes of the reflecting surface, the accuracy of the profile is improved.

Assume that the initial position of the reflector nodes of the cable net structure is:

(17) $$m_{0p}={\left[m_{0,i_{1}}^{T},m_{0,i_{2}}^{T},\cdots ,m_{0,i_{p}}^{T}\right]}^{T},$$

$m_{0,i_{p}}^{}$ (p = 1, 2, …, *n*) is the initial position vector of node *i*_p_, and *n* is the number of reflecting surface nodes.

*i*_p_ is the front cable net surface node, *i*_q_ is the rear cable mesh surface node. The length of the shape memory alloy wires in the initial equilibrium state can be expressed as:

(18) $$L^{s}={\left[\left(x_{i_{p}}-x_{i_{q}}\right)+\left(y_{i_{p}}-y_{i_{q}}\right)+\left(z_{i_{p}}-z_{i_{q}}\right)\right]}^{1/2}.$$

After prestressing, the length of the shape memory alloy wire becomes:

(19) $$L_{0}^{s}=\frac{L^{s}}{1+\varepsilon_{0}^{s}},$$

where the initial strain is:

(20) $$\varepsilon_{0}^{s}=\varepsilon_{L}\xi_{0}+\frac{\sigma_{0}^{s}}{E^{s}A^{s}},$$

where $\varepsilon_{L}$ is the maximum residual strain of the shape memory alloy wire. The initial martensite content $\xi_{0}$ and initial stress $\sigma_{0}^{s}$ are expressed as:

(21) $$\xi_{0}=\frac{1}{2}\cos\left\{\frac{\pi }{\sigma_{s}^{cr}-\sigma_{f}^{cr}}\left[\sigma_{0}^{s}-\sigma_{f}^{cr}-C_{M}\left(T-M_{s}\right)\right]\right\}+\frac{1}{2},$$

(22) $$\sigma_{0}^{s}=\frac{F_{0}^{s}}{A^{s}}.$$

The displacement vector of node *i*_p_ in the adjustment process is expressed as:

(23) $$\Delta D_{ip}={\left[\Delta x_{i_{p}},\Delta y_{i_{p}},\Delta z_{i_{{}_{p}}}\right]}^{T}.$$

The displacement vector of cable net structure deformation during adjustment can be written as:

(24) $$\Delta D_{p}={\left[\Delta D_{0,i_{1}}^{T},\Delta D_{0,i_{2}}^{T},\cdots ,\Delta D_{0,i_{p}}^{T}\right]}^{T}.$$

Make the shape memory alloy wire have a strain corresponding to the adjustment amount, which can be expressed as:

(25) $$d_{1}-d_{2}={\left(\Delta x_{i_{p}}^{2}+\Delta y_{i_{p}}^{2}+\Delta z_{i_{p}}^{2}\right)}^{1/2}.$$

The node position after adjustment can be expressed as:

(26) $$m_{p}=m_{0p}+\Delta D_{p}.$$

### 2.2. Model Parameters

#### 2.2.1. Material Parameters

**Figure 4:**
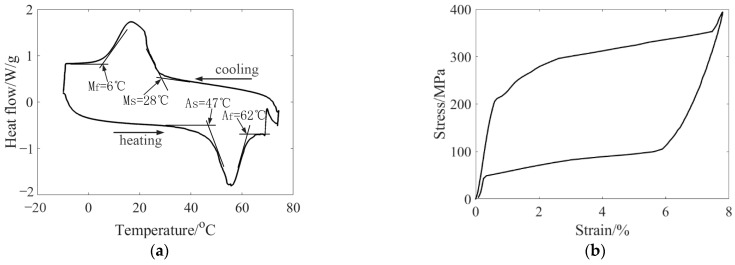
SMA parameter test (**a**) DSC test curve; (**b**) 75 °C Stretch curve.

The shape memory alloy wires used in the experiment were 0.25 mm in diameter and were produced by Jiangyin Pell Technology Co., Ltd, Wuxi, China. The material was tested by DSC and DMA to obtain accurate phase transformation parameters. The DSC curve of the shape memory alloy wire is shown in Figure 4a. The starting temperature and ending temperature of austenite phase transformation are obtained from the tangent intersection point of the curve during the heating process. The starting temperature and ending temperature of martensite transformation can be obtained from the tangent intersection point of the curve during the cooling process.

The shape memory alloy wire was stretched and unloaded at different temperatures in a closed environment, and the relationship between stress and strain and temperature was obtained. The material parameters of the shape memory alloy are calculated through multiple sets of curves. Figure 4b shows the tensile curve of the shape memory alloy wire at 75 °C. The material parameters obtained from the test are shown in Table 1.

#### 2.2.2. Structural Parameters

**Figure 5:**
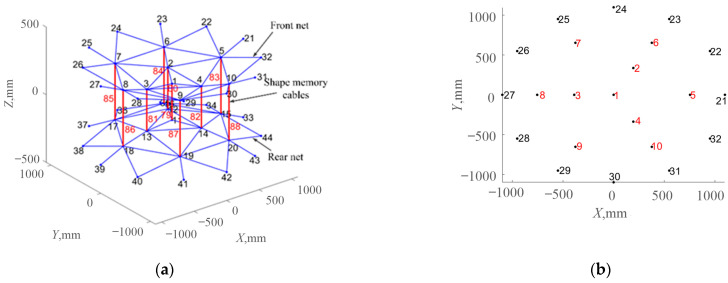
Shape memory cable net structure model (**a**) Experimental model; (**b**) Nodes number and position of the front cable net.

The shape memory cable net structure is shown in Figure 5a, which is composed of a front cable net, rear cable net, vertical cable and edge truss. With a diameter of 2.2 m, there are 88 cables in total, of which the vertical cables numbered 79–88 are shape memory alloy wires. The detailed parameters of the cable net structure are shown in Table 2. The position and number of nodes on the front cable net are shown in Figure 5b. Nodes 1 to 10 are free nodes. They are connected with cable segments, and their displacement and rotation are not constrained by external forces. Nodes 21 to 32 are fixed nodes, which are fixed on the surrounding truss.

The cable length and prestress of the shape memory cable net structure under the initial equilibrium state are calculated by the traditional form-finding and force-finding method. The maximum prestress of the front cable net and the rear cable net is 34.21 N, and the minimum prestress is 10.69 N. As the vertical cable, the shape memory alloy wire is the first circle in the centre of the reflecting surface, and the second and third circles are outwards in turn. The length of the first circle of the cable is 267.08 mm, and the prestress is 3.8 N. The length of the second circle of the cable is 334.81 mm, and the prestress is 7.15 N. The length of the third circle of the cable is 411.63 mm, and the prestress is 12.49 N.

## 3. Active Adjustment of the SMC Structure at Room Temperature

### 3.1. Normal Temperature Protocol

**Figure 6:**
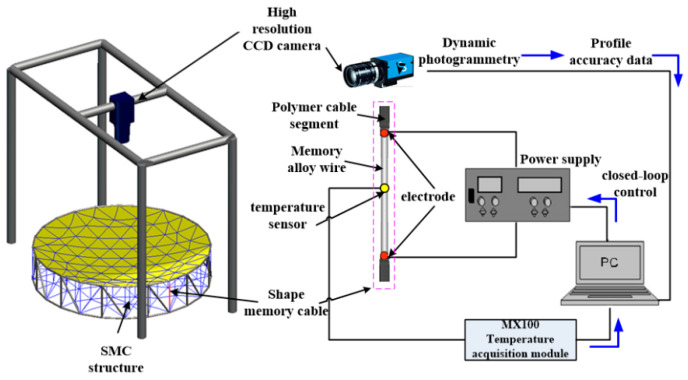
Schematic diagram of the normal temperature test scheme.

The shape memory cable net structure is actively adjusted at room temperature to verify its effectiveness in adjusting the precision of the reflector surface. Active adjustment uses the external power supply to heat the shape memory alloy wire to make a phase transformation to adjust the surface accuracy. During the heating process, the shape memory cable undergoes austenite phase transformation, and the strain decreases, making the vertical cable tight. When the power supply is turned off, the shape memory cable gradually cools down, martensitic transformation occurs, the strain gradually increases until the original length is restored, and the vertical cable tension gradually decreases. The experimental scheme is shown in Figure 6.

**Figure 7:**
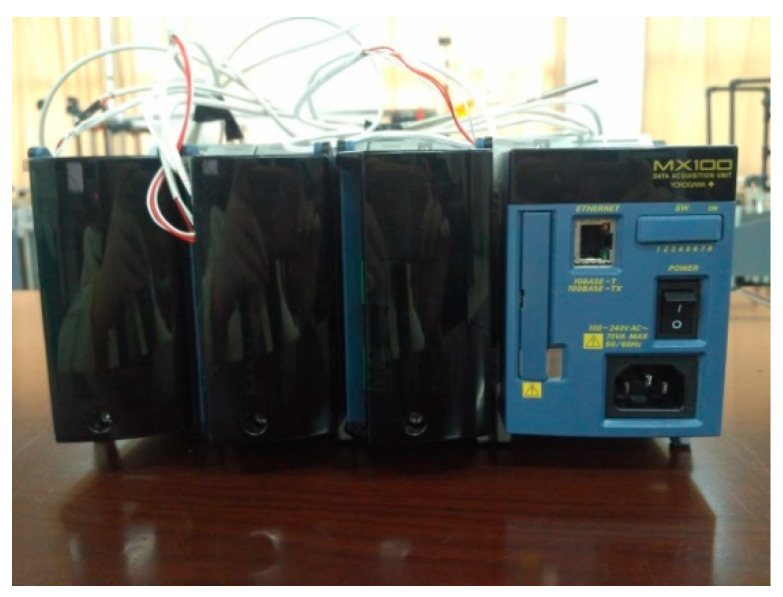
Temperature acquisition equipment.

**Figure 8:**
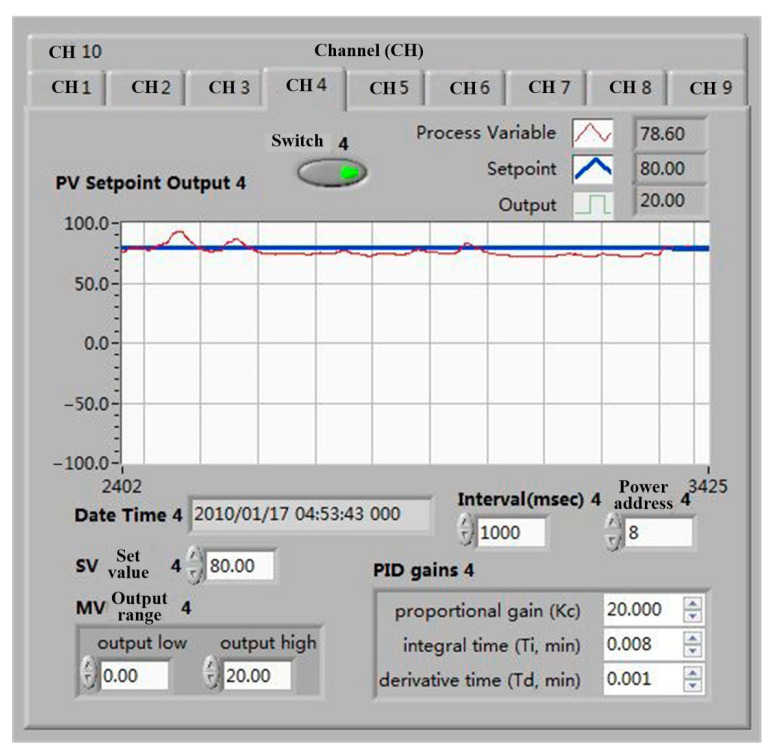
Temperature control interface.

During the experiment, temperature and profile accuracy (3D coordinates of reflector nodes) were two data points to be collected. The MX100 multichannel data acquisition system of Yokogawa Corporation of Japan was selected as the temperature acquisition equipment, as shown in Figure 7. Through PID control, the temperature error can meet the temperature requirements of shape memory alloy wire phase transformation, as shown in Figure 8. When the set temperature does not meet the specified requirements, the current flowing through the memory alloy wire is increased through closed-loop control, and the temperature is continuously increased. When the temperature reaches the set value, the temperature is kept stable.

**Figure 9:**
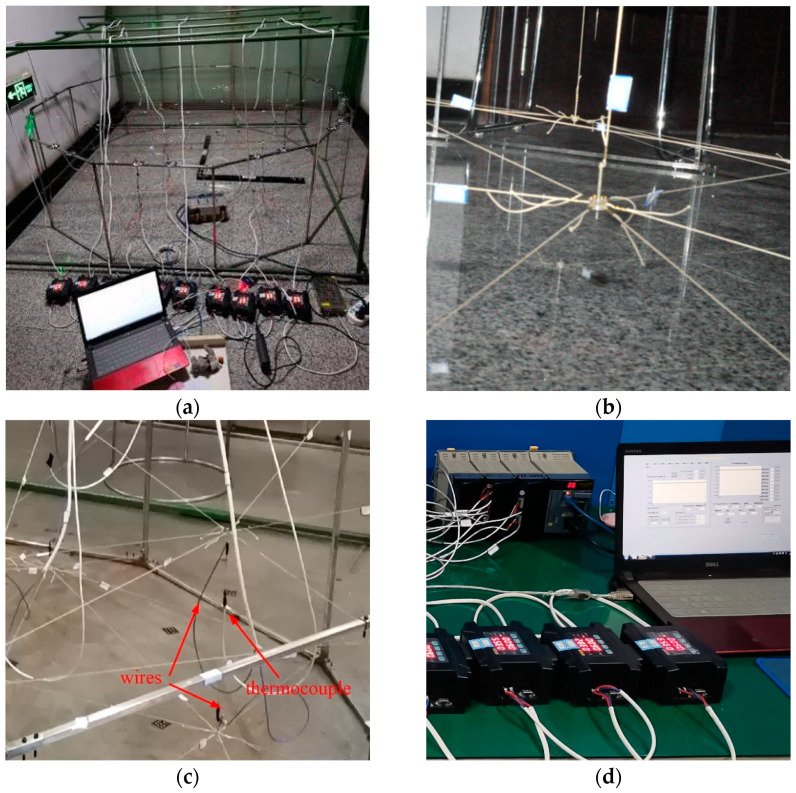
Normal temperature experiment: (**a**) Field test; (**b**) Connection mode; (**c**) Wires and thermocouples; (**d**) Controllable power supply.

The field test is shown in Figure 9. Figure 9a shows the overall structure diagram. Figure 9b shows the connection of the reflective surface and vertical cable with adjustable bolts. Figure 9c shows the wires and thermocouples. Figure 9d shows the power supply and MX100 temperature acquisition device.

The wire is connected at both ends of the SMA wire to provide current and heat the SMA wire. The thermocouple is attached to the SMA wire to provide temperature data. The wire is hung on the rack to eliminate the impact of its weight on the reflective surface.

Assuming that the heat generated during heating is Q, the heat consumed by convection with air is $Q_{1}$, and the heat absorbed by SMA wire is $Q_{2}$, the radiation heat dissipation of alloy wire is not considered; then,

(27) $$Q=Q_{1}+Q_{2}.$$

The current heat effect formula, convection heat dissipation formula and heat absorption formula of shape memory alloy wire are respectively expressed as:

(28) $$Q=I^{2}Rdt,$$

(29) $$Q_{1}=kS\Delta Tdt,$$

(30) $$Q_{2}=CmdT.$$

According to the above formulas, the following formula can be obtained:

(31) $$dt/dT=Cm/\left(I^{2}R+kS\Delta T\right).$$

The resistance of the shape memory alloy wire can be expressed as

(32) R=ρl/A,

where I is the current, R is the resistance value of SMA wire, ρ is the resistivity and its value is 10^−6^ Ω·m, *l* is the length, and A is the cross-sectional area. C is the specific heat capacity, and its value is 550 J/(kg·K), m is the mass, k is the convective heat dissipation coefficient, S is the surface area, and ΔT is the temperature change. The heating current and time can be determined by the above formulas and the phase transformation temperature of SMA.

In the experiment, the node coordinates were collected using the Tianyuan photogrammetric system. The measuring system includes computers, high-resolution cameras, reference rulers, coding points and marking points. When the photogrammetric system is used for measurement, the position information of all marked points can be obtained at the same time. To accurately identify the reflective surface nodes, the cable net structure is first photographed from different angles and positions to obtain multiple digital photos, and then the image is processed to obtain the three-dimensional spatial coordinates of the nodes.

During the heating process, the currents range between 1.3~1.5 A, and the voltages range between 4.6~7.2 V. When the temperature reaches the set value, the temperature is kept constant, and the node position of the reflector is obtained through photogrammetry. The temperature fluctuation range is 56.7~61.6 °C during shooting. The power was turned off after photographing, and the shape memory wires were gradually cooled to normal temperature.

### 3.2. Analysis of Normal Temperature Test Results

**Figure 10:**
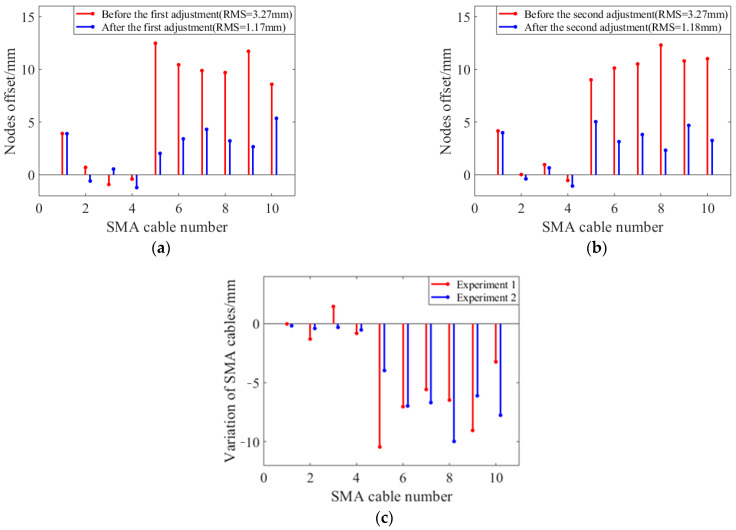
Normal temperature test results: (**a**) The node deviations in the first experiment; (**b**) The node deviations in the second experiment; (**c**) Comparison of cable length changes in two experiments.

Two groups of active adjustment experiments were carried out at room temperature to verify the ability of shape memory alloy wires to adjust the surface accuracy of the shape memory cable net structure. The node deviations of the reflector before and after adjustment are shown in Figure 10a and Figure 10b, respectively. The length changes of shape memory alloy wires during the first and second adjustment processes are shown in Figure 10c. The results show that the length of the vertical cable changes basically the same during the two adjustments, and the surface accuracy before and after the two adjustments is also basically consistent. The shape memory alloy wire adjustment ability is relatively stable.

According to the experimental results, the vertical cable deviation in the second circle is the smallest, while the deviation in the first and third circles is relatively large. During the adjustment process, the deformation of the first vertical cable is the smallest, and the deformation of the third vertical cable is the largest. The main reason is that the prestress of the first circle is the smallest, and the corresponding initial content of martensite is also the smallest. The third circle has the largest prestress, and the corresponding initial content of martensite is also the largest, so the strain during transformation is relatively large. After two experiments, the reflecting surface can basically return to its initial position, and the shape memory alloy wire has good stability.

**Figure 11:**
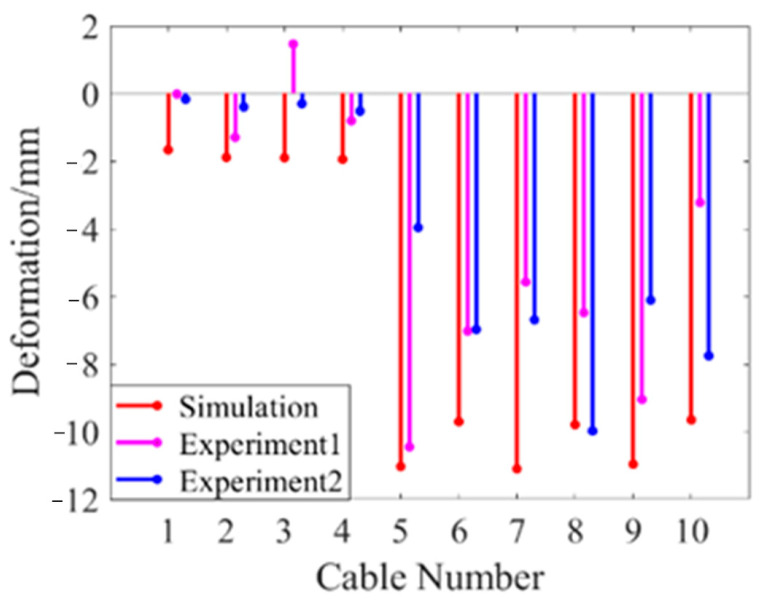
Deformation of SMA wires before and after optimization.

The shape memory cable net structure in the experiment is simulated by MATLAB, and the simulation results are compared with the experimental results. The comparison of surface accuracy and average deviation of nodes before and after adjustment is shown in Table 3, and the comparison of deformation of shape memory alloy wires is shown in Figure 11. The results show that under the same initial state, the experimental data are close to the simulation data, and the reliability of the model can be verified by experiments.

## 4. Experiment of SMC Structure under High and Low Temperatures

### 4.1. High and Low Temperature Experimental Scheme

**Figure 12:**
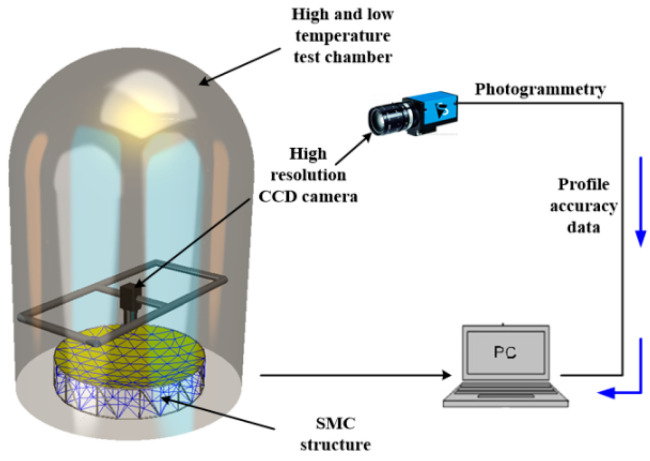
Schematic diagram of the high- and low-temperature experimental schemes.

**Figure 13:**
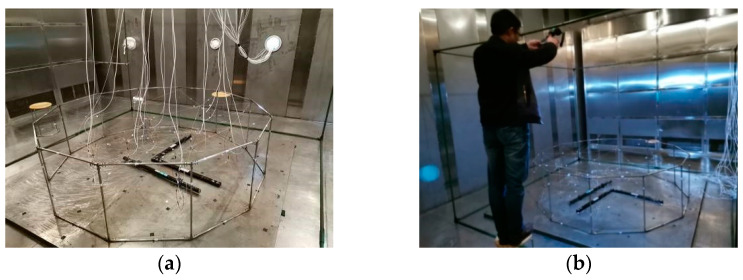
Field test of the high- and low-temperature experiments: (**a**) SMC structure; (**b**) Field test.

To verify the effectiveness of passive adjustment and the performance of active adjustment of the shape memory cable net structure in the radiation environment, high- and low-temperature test boxes are used to apply a temperature load on the shape memory cable net structure. During passive adjustment, the shape memory alloy wire does not need to be electrified and controlled. The rise and fall of ambient temperature can cause it to undergo reversed-phase transformation and phase transformation. During the heating process, the austenite phase transformation occurs when the temperature reaches $A_{s}$. During cooling, martensitic transformation occurs when the temperature reaches $M_{s}$. The experimental scheme is shown in Figure 12, and the experimental field test is shown in Figure 13.

**Figure 14:**
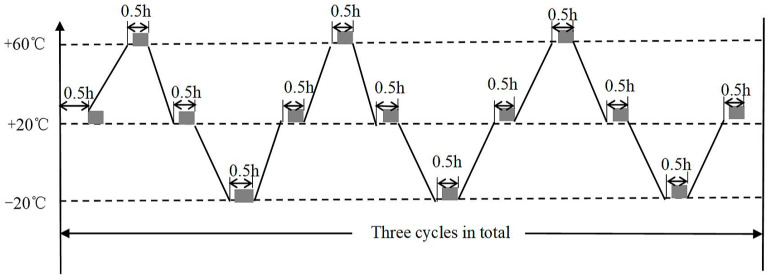
Schematic diagram of the experimental temperature cycle.

The parameters of the material in the experiment were obtained from the above experiments. A temperature environment of −20~60 °C was selected based on the normal operating temperature of the camera. The temperature was maintained for 30 min in the experiment to obtain stable measurement parameters. For comparison, a temperature load was applied to the normal cable net, passive adjustment of the shape memory cable net and active adjustment of the shape memory cable net. A high- and low-temperature box was used to provide a temperature environment of −20~60 °C. The temperature cycle diagram is shown in Figure 14. First, it is heated from 20 °C to 60 °C, then cooled to −20 °C, and finally heated to 20 °C to complete a cycle. It is then held for 30 min at 20 °C, 60 °C and −20 °C. Thermal deformation measurements are conducted in the holding stage.

### 4.2. Analysis of Experimental Results

**Figure 15:**
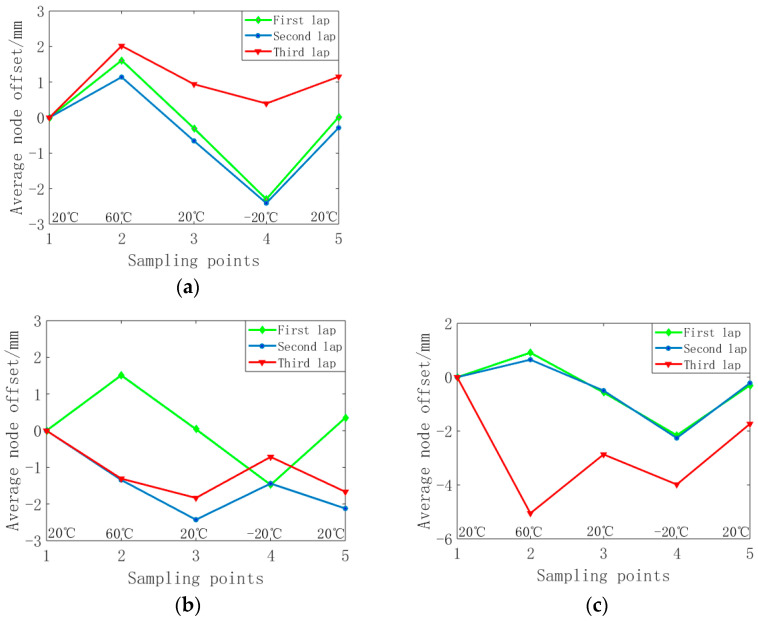
Average node offset per circle: (**a**) Ordinary cable net; (**b**) Passive adjustment; (**c**) Active adjustment.

During the experiment, the change in node position with temperature is shown in Figure 15. At 60 °C and −20 °C, the displacement of nodes is the largest and that of common cable net nodes is relatively small. When the shape memory cable net is actively adjusted, the displacement of the node is large, and the adjustment of the surface accuracy is large. The displacement of nodes close to the truss is the largest, which is due to the maximum prestress of these nodes.

**Figure 16:**
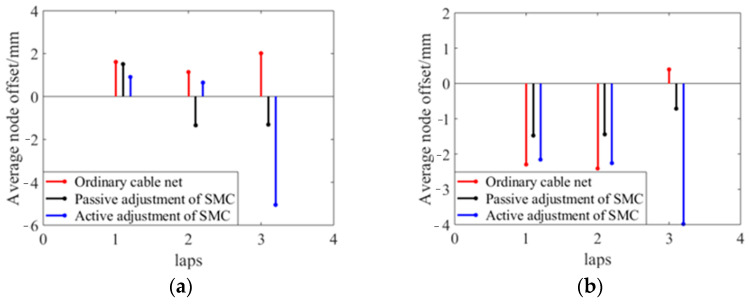
Comparison of average node offset: (**a**) At 60 °C; (**b**) At −20 °C.

We collected the node coordinates of the reflector at 60 °C and −20 °C, calculated the average value of the node offset of each circle, and analysed the node deviation from the initial state. The results are shown in Figure 16a,b. It can be seen in the figure that due to the thermal expansion and contraction characteristics of the material, the nodal point deviates from the initial state in the opposite direction at 60 °C and −20 °C. The node displacement of the first circle is close to that of the second circle, while the node displacement of the third circle is relatively large. This is related to the large initial prestress of the third vertical cable. Due to the complex interaction of reflector nodes, some nodes do not fully follow the rules.

**Figure 17:**
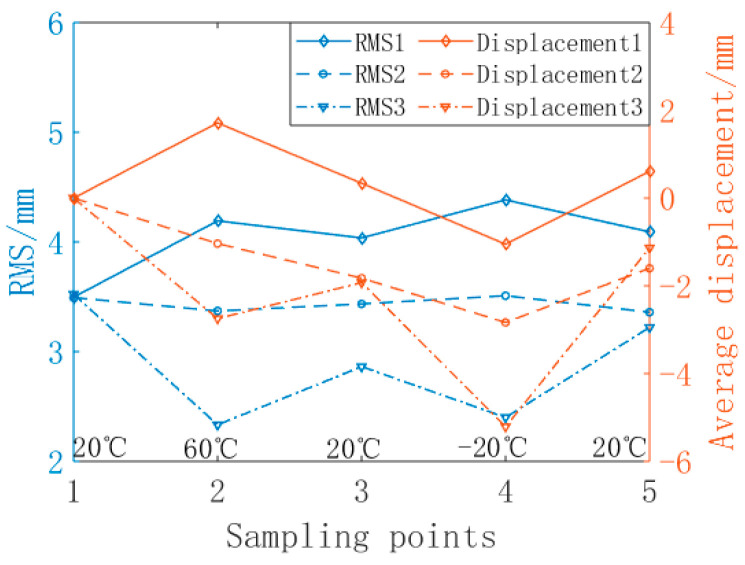
Comparison of RMS changes and average displacement of nodes under high- and low-temperature cycles.

The measurement results are affected by the levelness of the antenna base in high- and low-temperature experiments. During the analysis, the data are processed with error to reduce the impact of the external environment. Figure 17 shows the variation in the surface accuracy of the cable net structure with temperature and the average displacement of nodes during the temperature cycle.

(1) At 60 °C, the error of the reflective surface of the ordinary cable net increases obviously. Due to the creep effect of the high- and low-temperature environment on the polymer cable, the precision of the common cable net profile does not return to the initial state when the temperature cycle returns to 20 °C. Its surface accuracy increased from the initial 3.50 mm to 4.38 mm at most.
(2) The surface accuracy of the shape memory cable net structure is relatively stable, and the variation is small when it is adjusted passively. The initial surface accuracy is 3.49 mm, and it becomes 3.36 mm at the end of a cycle.
(3) When the shape memory cable net structure is actively adjusted, the surface accuracy is significantly improved at both 60 °C and −20 °C. The initial surface accuracy is 3.52 mm, which becomes 2.33 mm at 60 °C and 2.40 mm at −20 °C under active adjustment. The surface accuracy at the end of one cycle becomes 3.22 mm.
(4) At 60 °C, compared to ordinary cable net structures, the passive adjustment improves the surface accuracy by 19.6%, while the active adjustment improves it by 44.4%. At −20 °C, compared to ordinary cable net structures, the passive adjustment improves the surface accuracy by 20.1%, while the active adjustment improves it by 45.2%.

Compared with the state at the beginning and the end of the temperature cycle, the shape memory cable net structure has a small change in surface accuracy and has a certain shape preservation ability under the temperature load.

The model used in the experiment has not yet taken into account the effects of creep and thermal ratcheting on the materials. Due to factors such as creep, there are some differences between experimental and simulation results in high- and low-temperature experiments. In later research, the effects of creep and thermal ratcheting will be considered to make the experimental environment closer to the space environment.

## 5. Conclusions

A method of surface adjustment for a cable net structure using shape memory alloy wire is analyzed in this paper. The shape memory cable (SMC) net structure is simulated and tested. The effectiveness of the shape memory alloy in adjusting the surface accuracy of the cable net structure in a thermal radiation environment is verified. The following conclusions can be drawn from the study.

(1) The change in stress and strain during the phase transformation of the shape memory alloy wire can adjust the surface accuracy of the cable net structure.
(2) In normal temperature and high- and low-temperature environments, the surface accuracy of the cable net structure has been improved through the adjustment of the shape memory alloy wire. Compared with ordinary cable net, at 60 °C and −20 °C, surface accuracy of SMC structure has improved by 44.4% and 45.2%, respectively.
(3) Compared with passive adjustment, active adjustment has a larger adjustable amount of surface accuracy. The applicable temperature range is larger, but an external power supply is needed.

Shape memory alloy wire is used to actively and passively adjust the reflective surface of the cable net structure. The proposed passive adjustment method converts adverse factors such as thermal radiation into favorable factors for the on-orbit adjustment of surface accuracy. These research results have reference value for practical engineering applications.

Wang utilized PZT actuators to improve the surface accuracy of cable net structures. During the optimization process, an external high-voltage power supply of 347.7 V was required, which is not conducive to practical engineering implementation [20]. In the scheme of this paper, the maximum power supply for active regulation is 7.2 V, and passive regulation does not require an external power supply, which is more conducive to engineering implementation.

In the current plan, there is sometimes a problem of excessive adjustment in the connection method of vertical cables, resulting in a reverse increase in RMS. Later research can adopt a parallel approach to control the adjustment amount of SMA vertical cables. At the same time, using only one SMA material limits the temperature range. In later research, SMA materials with different phase transition temperatures can be considered to operate at high and low temperatures, respectively.

## Figures and Tables

**Table 1:** SMA phase transformation parameters.

Parameters	Values	Parameters	Values
$M_{s}$/°C	28	$A_{s}$/°C	47
$M_{f}$/°C	6	$A_{f}$/°C	62
$E_{M}$/GPa	16.98	$E_{A}$/GPa	45.32
$C_{M}$/MPa/°C	8.14	$C_{A}$/MPa/°C	11.27

**Table 2:** Parameters of the SMC structure.

Parameters	Values	Parameters	Values
Diameter/mm	2200	Elastic modulus of polymer cables/GPa	20
Number of polymer cables	88	Diameter of polymer cables/mm	1
Number of free nodes	20	Number of SMA cables	10
Number of fixed nodes	24	Diameter of SMA cables/mm	0.25

**Table 3:** Error comparison between simulation and experiment.

Items	Initial RMS/mm	Optimized RMS/mm	Initial Average Deviation/mm	Optimized Average Deviation/mm
Simulation	3.278	1.177	6.764	1.948
Experiment 1	3.275	1.170	6.607	2.358
Experiment 2	3.273	1.181	6.831	2.542

## Data Availability

The data presented in this study can be requested from the corresponding author.
